# Non-12α-Hydroxylated Bile Acids Improve Piglet Growth Performance by Improving Intestinal Flora, Promoting Intestinal Development and Bile Acid Synthesis

**DOI:** 10.3390/ani13213380

**Published:** 2023-10-31

**Authors:** Jianwei Qin, Xinke Wei, Mingming Cao, Baoming Shi

**Affiliations:** College of Animal Science and Technology, Northeast Agricultural University, Harbin 150030, China; qin1467453758@163.com (J.Q.); ak13263621369@163.com (X.W.); caomingming1999@163.com (M.C.)

**Keywords:** bile acid metabolism, weaned piglets, gut microbiota, growth performance, intestinal development

## Abstract

**Simple Summary:**

Bile acids have various physiological functions in the body. On the one hand, they can promote the digestion and absorption of fats as emulsifiers, and on the other hand, they participate in many metabolic pathways as signaling molecules. In addition, bile acids can shape the gut flora and improve gut health. This experiment aimed to investigate the effects of non-12α-hydroxylated bile acids on the growth and development, and intestinal flora of weaned piglets. This study showed that the addition of non-12α-hydroxylated bile acids could improve growth performance and nutrient digestibility in weaned piglets without negatively affecting the liver. In addition, the addition of non-12α-hydroxylated bile acids also improved the intestinal flora, increased the abundance of *Lactobacillus_johnsonii*, and increased the content of short-chain fatty acids.

**Abstract:**

As an emulsifier and bioactive substance, bile acids (BAs) participate in the absorption of nutrients and in various physiological processes. The objective of this experiment was to investigate the effects of non-12α-hydroxylated BAs (including hyocholic acid, hyodeoxycholic acid and chenodeoxycholic acid, from now on referred to as NBAs) on growth performance, BAs metabolism and the intestinal flora of piglets. The experiment included four groups, with eight piglets per group. The four groups of pigs were fed 0, 60, 120 and 180 mg/kg of NBAs, respectively. The results show that adding NBAs significantly increased the final weight (FW), average daily feed intake (ADFI), average daily gain (ADG), and digestibility of crude fat (EE) and organic matter (OM) in piglets (*p* < 0.05). Adding NBAs significantly increased the villus height (VH) of the jejunum and ileum (*p* < 0.05). In addition, NBAs supplementation increased the content of urea nitrogen (BUN) and creatinine (CREA) as well as the ratio of urea nitrogen to creatinine (BUN/CREA) in serum (*p* < 0.05). Adding NBAs can affect the genes related to BAs enterohepatic circulation. Specifically, adding NBAs significantly decreased the relative mRNA abundance of *FXR* in the liver (*p* < 0.05), significantly increased the relative mRNA abundance of *CYP27A1* (*p* < 0.05), and significantly increased the relative mRNA abundance of *NTCP* (*p* < 0.05). Adding NBAs also significantly decreased the relative mRNA abundance of *FXR* in the ileum (*p* < 0.05). In the full-length 16S rDNA sequencing analysis, ten biomarkers were found from the gate to the species level. NBAs mainly enriched *Lactobacillus_Johnsonii* and decreased the abundance of *Streptococcus_alactolyticus*. Short-chain fatty acids (SCFAs) content in the colon was significantly increased (*p* < 0.05). These results indicate that NBAs supplementation can improve the growth performance of piglets, promote the development of the bile acid replacement pathway and improve intestinal flora.

## 1. Introduction

In commercial production, piglets are usually weaned at around 3–5 weeks, and weaning is challenging for piglets. At the same time, the development of bile acid (BA) synthase in piglets is still in the growth phase from 21 days to 49 days of age [1]. Limited BA secretion and an immature digestive system are the main causes of insufficient lipid digestion ability in young animals [2]. Weaning can induce VH shortening and CD increase, and changes in the small intestine’s structure and function can affect the intestine’s nutrient absorption capacity and barrier function [3]. The change in piglets’ diet can easily lead to reduced feed intake and slow growth of piglets. Therefore, adding a substance that can improve the lipid digestion ability of piglets and promote intestinal development is necessary, and BAs meet this requirement. Porcine BAs mainly include hyodeoxycholic acid (HDCA), chenodeoxycholic acid (CDCA) and hyocholic acid (HCA). HCA and its derivatives account for about 76% of the BAs pool of pigs, and the resistance of pigs to some metabolic diseases is related to HCA and its derivatives [4]. Studies have shown that adding BAs to the diet of laying hens can promote fat absorption, improve intestinal flora and increase the laying rate [5]. Supplementation of BAs in piglets’ diets can promote intestinal development and improve intestinal flora [6]. The above studies show that adding BAs can achieve good results in animal production.

BAs are sterols produced by animal cholesterol metabolism and are divided into primary and secondary BAs. BAs can be divided into 12α-hydroxylated BAs and non-12α-hydroxylated BAs based on the presence or absence of hydroxyl (-OH) groups at the C12 position. The 12α-hydroxylated BAs mainly include cholic acid (CA), deoxycholic acid (DCA) and ursocholic acid (UCA), while the non-12α-hydroxylated BAs include CDCA, HCA and HDCA. Studies have shown that an increased 12α-OH BAs ratio leads to a lost ability to control lipid homeostasis and inflammation [7]. The HCA, HDCA and CDCA added in this experiment are all types of NBAs and have application value. BAs are essential in synthesizing fatty acids, lipids and lipoproteins [8]. BAs are also amphoteric molecules containing hydrophilic (hydroxyl, carboxyl) and hydrophobic (alkyl) groups, which have surface active functions and can directly act as emulsifiers in the three stages of fat emulsification, digestion and absorption [9]. BAs can break down fats into tiny lipid droplets to increase the contact area with lipase and promote the digestion and absorption of lipids and fat-soluble vitamins in chyme [10]. In our previous study, we found that adding emulsifiers increased nutrient digestibility and pancreatic lipase activity in weaned piglets and promoted the growth and development of piglets [11]. BAs are also involved in regulating glucose homeostasis, energy homeostasis, and immunity [12,13]. In addition, BAs can increase the abundance of beneficial bacteria in the gut, such as *Lactobacillus* and *Faecalibacterium* [14]. BAs can also promote intestinal organoid growth, increase intestinal epithelium proliferation, and promote intestinal stem cell renewal and damage regeneration [15].

This study investigated whether adding NBAs affected growth performance, nutrient digestibility, intestinal development, intestinal flora and SCFAs in weaned piglets. In addition, in order to determine whether the dosage of added NBAs was within the safe range and whether the addition of NBAs promoted the synthesis and secretion of BAs, we further examined the blood biochemical indicators and the expression levels of genes related to BA synthesis and transport.

## 2. Materials and Methods

Animal care and treatment complied with the Laboratory Animal Management Regulations (revised 2016) of Heilongjiang Province, China, and the Animal Welfare Committee protocol (# NEAU-2013) of Northeast Agricultural University.

### 2.1. Animals, Diets and Treatments

Thirty-two 35-day-old piglets (Duroc × (Landrace × Yorkshire)) with a similar parity and a body weight of 10.15 ± 0.38 kg were randomly assigned to four groups (50% male and 50% female). The supplemental NBA levels in the four groups were 0, 60, 120 and 180 mg/kg, respectively. The experiment lasted for 28 days. The nutritional level and composition of the base diet of piglets (Table S1) met the requirements of the NRC. The experiment was conducted in Acheng District, Heilongjiang province, in pig houses with temperatures above 15 °C. During the experiment, the enclosures were disinfected as per the epidemic prevention requirements, and the experimental piglets were raised in individual metabolic cages (1.78 × 0.84 × 1.40 m). During the experiment, the piglets were fed thrice daily and were free to drink and eat. The NBAs used in this study included HCA ≥ 36.46%, HDCA ≥ 24.18% and CDCA ≥ 16.15%, all of which were non-12-OH BAs provided by Harbin Dongmu Biotechnology Co., Ltd. (Harbin, China).

### 2.2. Sample Collection and Preparation

All piglets were fasted overnight, weighed after corona treatment at 5:00 on day 29, and then slaughtered. The blood of each piglet was collected into a heparin sodium test tube through collection from the anterior vena cava and then centrifuged for 15 min (rotation speed 3000× *g*). The upper serum was collected, stored at 4 °C, and then transferred to −40 °C until testing. A section of small intestine tissue was rinsed with normal saline and placed in a 50 mL spiral tube (containing 4% paraformaldehyde) for preservation away from light. The tissue samples were stored in the refrigerator at −80 °C.

### 2.3. Growth Performance and Nutrient Digestibility

The piglets were weighed before testing and slaughter. The remaining feed of the piglets was weighed and recorded daily before feeding to obtain ADFI and used to calculate the other growth parameters. Piglet feces were put into sealed plastic bags, treated with 10% sulfuric acid for nitrogen fixation, and immediately stored in a −20 °C refrigerator until testing. The contents of total energy (GE), crude protein (CP), crude fiber (CF), crude fat (EE) and organic matter (OM) in the feces and diets were determined, and the nutrient digestibility of each nutrient was calculated [16].

### 2.4. Plasma Biochemical Analysis

The contents of urea nitrogen (BUN), creatinine (CREA), urea nitrogen/creatinine (BUN/CREA), alkaline phosphatase (ALP), gamma-glutamyl transferase (GGT), total BAs (TBA) and total bilirubin (TBIL) in plasma samples were determined by an automatic biochemical analyzer (Mindary BS−2000 M2, Shenzhen, China).

### 2.5. Intestinal Morphology Analysis

The intestinal histomorphology of the weaned piglets was observed by hematoxylin-eosin (HE) staining. When slicing, at least two wax slices with a thickness of 5 µm were cut consecutively for subsequent experiments. The prepared sections were scanned using a SCI-Digital Section Scanning System (Jinan, China), and at least 3 different typical fields were selected in ZYFviewer 2.0.1.5 (Jinan, China) to take photos to record the development of the jejunum and ileum. Intestinal villus height (VH) and crypt depth (CD) were measured using Image J 1.53t.

### 2.6. Quantitative Real-Time PCR

Total RNA was extracted from the liver using an RNA extraction Kit (E.Z.N.A. ^®^ Total RNA Kit I, R6834−01, Omega, Beijing, China), and A260/A280 values were measured using an ultramicro spectrophotometer to ensure the ratio was between 1.8 and 2.0. The PrimeScript^TM^ RT Reagent Kit (RR047A, TaKaRa, Beijing, China) was used for RNA reverse transcription, and the RT-PCR reaction was performed using the TB Green^®^ Premix EX Taq^TM^ (RR420A, TaKaRa, Beijing, China) kit [17]. The RT-PCR primers were provided by Sangon Biological (Shanghai) Co., Ltd. (Shanghai, China). The design and synthesis primer sequences are shown in Table S2.

### 2.7. 16S rDNA

The total DNA of 0 and 180 mg/kg NBA groups was extracted by the CTAB method, and the DNA quality was detected by agarose gel electrophoresis (1%). PCR amplification was performed with universal primers. The synthesized primers contained specific barcode sequences to distinguish samples. After 2% agarose gel electrophoresis, the target fragment was recovered and purified. The recovered amplified products were quantified using the QuantiFluor^TM^-ST Blue fluorescence quantification system (Promega, Madison, WI, USA) and then proportionally mixed. The sequencing libraries of the mixed products were prepared using Pacific Biosciences SMRTbell Template Prep kit 1.0 (Menlo Park, CA, USA) and sequenced by computer.

### 2.8. Quantification of SCFAs

A 2 g sample of colon contents and 2 mL of ultrapure water were added to a 10 mL screw tube. They were extracted for 48 h and then centrifuged at 6000× *g* for 10 min. The supernatant was kept and centrifuged at 10,000× *g* for 10 min. The supernatant was retaken and washed with a 0.22 μm cellulose membrane filter at 10,000× *g* for 10 min after filtration with a membrane filter. The supernatant was retained and filtered with a 0.22 μm cellulose membrane. The filtered liquid was stored at 4 °C. The gas chromatograph used was GC-2010 (Shimadzu Corporation, Tokyo, Japan). The carrier gas was nitrogen. The SPL1 temperature was set at 230 °C. The pressure was 90 kPa. The column oven temperature was 180 °C. The FID temperature was 240 °C. The makeup flow rate was 30 mL/min.

### 2.9. Statistical Analysis

All data were tested for normality. SPSS 25 (IBM-SPSS Inc., Chicago, IL, USA) statistical software was used to perform one-way ANOVA, and linear and quadratic regression analyses on data other than the 16S rDNA data. Duncan’s method was used to make multiple comparisons. GraphPad Prism 8 was used to illustrate the test results. *p* ≤ 0.05 was considered a significant difference.

## 3. Results

### 3.1. Effects of NBAs on the Growth Performance of Piglets

From Table 1, we can see the effect of NBAs on the growth performance of piglets. Compared with the control group, the supplementation of NBAs linearly increased FW, ADFI and ADG (*p* < 0.05), but supplementation with NBAs had no significant effect on ADFI/ADG (F/G) (*p* > 0.05). 

### 3.2. Effect of NBAs on Nutrient Digestibility in Piglets

The nutrient digestibility of each nutrient is shown in Table 2. Adding NBAs linearly increased the digestibility of OM and EE (*p* < 0.05). The digestibility of EE and OM with 180 mg/kg NBAs was the highest. The additional level of NBAs had no significant effects on the nutrient digestibility of GE, CP and CF (*p* > 0.05).

### 3.3. Effects of NBAs on Blood Biochemistry of Piglets

The blood biochemical indicators are shown in Table 3. The values of BUN, CREA and BUN/CREA also increased linearly with the increase in NBAs supplemental level (*p* < 0.05). Adding NBAs had no significant effects on TBA, TBIL, ALP or GGT (*p* > 0.05).

### 3.4. Effects of NBAs on Intestinal Morphology of Piglets

**Figure 1:**
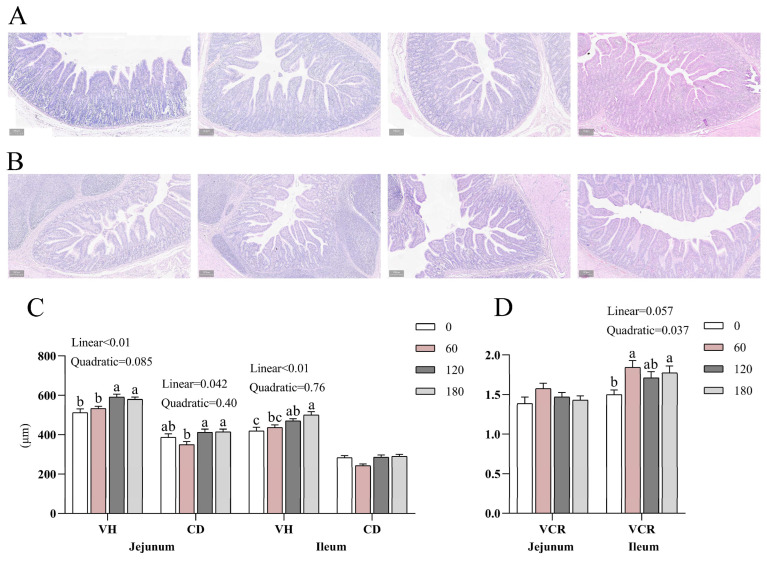
Effects of NBAs on intestinal morphology of jejunum and ileum. (**A**,**B**) Representative micrographs of fixed sections of jejunum and ileum with NBAs additions of 0, 60, 120, and 180 mg/kg from left to right. (**C**,**D**) VH: Villus height, CD: Crypt depth, VCR: VH/CD. Different superscript letters indicate significant differences (*p* < 0.05). *n* = 5.

Figure 1A–D shows representative photomicrographs of the jejunum and ileum of piglets supplemented with NBAs. Adding NBAs linearly increased the VH of the jejunum and ileum (*p* < 0.05). Adding NBAs linearly increased jejunum CD (*p* < 0.05). With the increase in NBAs, jejunum CD first decreased and then increased, and the value of jejunum CD was the smallest when 60 mg/kg of NBAs was added. In addition, adding 60 and 180 mg/kg NBA supplementation significantly quadratically increased ileum VH/CD (VCR) (*p* < 0.05).

### 3.5. Effects of NBAs on Relative mRNA Abundance of BA Cycling-Related Genes

**Figure 2:**
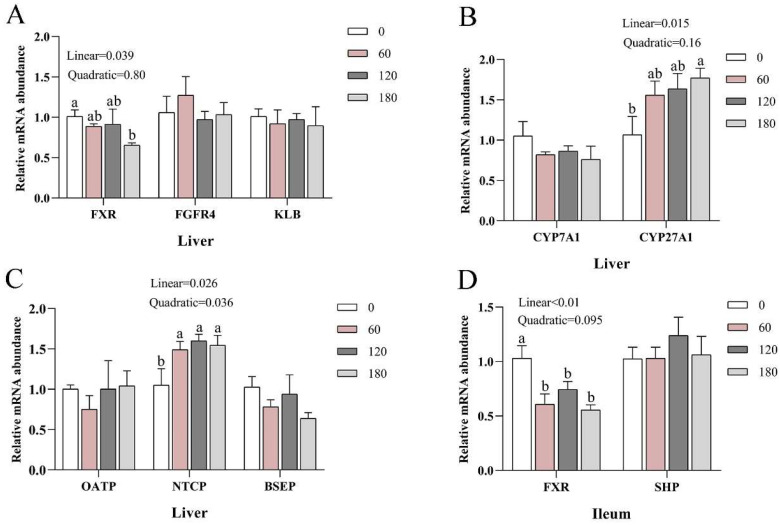
Effects of NBAs addition on the relative mRNA abundance of BA cycling-related genes in the liver (**A**–**C**). The effects of NBAs supplementation on the relative mRNA abundance of *FXR* and *SHP* in the ileum (**D**). Different superscript letters indicate significant differences (*p* < 0.05). In the quantitative real-time PCR test, the number of replicates was 5.

Figure 2A–D shows the effects of NBAs addition on the relative mRNA abundance of BA cycling-related genes in the liver. NBAs supplementation linearly decreased the relative mRNA abundance of farnesol X receptor (*FXR*) in the liver (*p* < 0.05), linearly increased the relative mRNA abundance of cytochrome P450 27A1 (*CYP27A1*) in the liver (*p* < 0.05), and linearly and quadratically increased sodium taurocholate co-transporting polypeptide (*NTCP)* in the liver (*p* < 0.05). NBAs supplementation linearly decreased the relative mRNA abundance of *FXR* in the ileum (*p* < 0.05). NBAs supplementation had no significant effects on the relative mRNA abundance of recombinant fibroblast growth factor receptor 4 (*FGFR4*), klotho beta (*KLB*), cytochrome P450 7A1 (*CYP7A1*), organic anion transporting polypeptide family (*OATP*), bile salt output pump (*BSEP*) or small heterodimer partner (*SHP*) (*p* > 0.05). 

### 3.6. Effects of NBAs on Colon Microbiota

**Figure 3:**
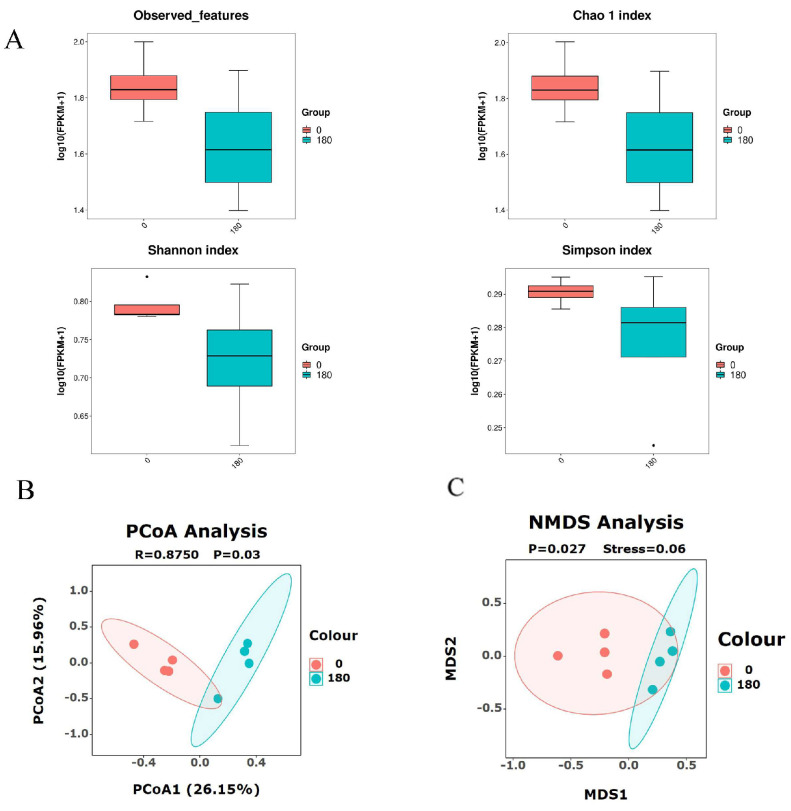
(**A**) Effects of NBAs on alpha diversity of colonic flora. (**B**) Effects of NBAs on PCoA analysis. (**C**) Effects of NBAs on NMDS analysis. *n* = 4.

The α and β diversity of colon microbiota is shown in Figure 3A–C. We found that comparing diets without to diets with the highest level of NBAs had no significant effect on the indexes for flora evenness and abundance of Chao1, Observed species, and Shannon and Simpson (*p* > 0.05). Adding NBAs had significant effects on the analysis of PCOA and NMDS (*p* < 0.05), indicating that NBAs affected species composition between groups, and further analysis of the differences in flora composition is needed.

**Figure 4:**
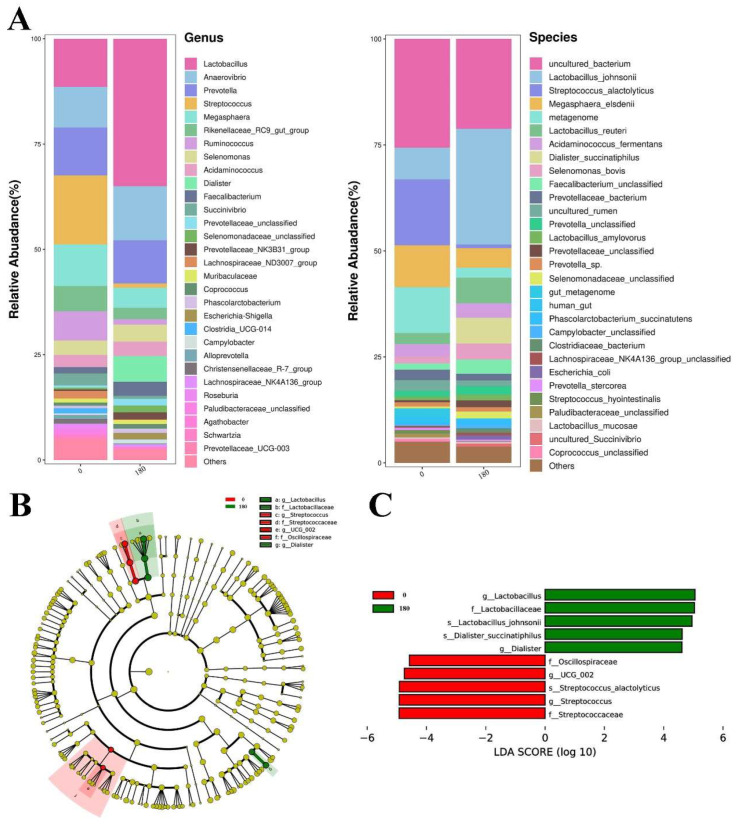
(**A**) Columnar stacking of colon flora at genus and species levels, and showing the top 30 species with the highest content. (**B**) LEfSe analysis from phylum level to genus level (LDA score ≥ 3.0) representing taxonomy levels from phylum to genus from inside to outside. Different color nodes represent microbial groups significantly enriched in the corresponding treatment groups. (**C**) The LDA scores of colon flora were compared from the phylum level to the species level, and the length of the bar graph represents the contribution of different species. *p* < 0.05 and LDA score (log10) > 3.0 were considered significant. *n* = 4.

The columnar stack diagram and linear discriminant analysis effect size (LEfSe) analysis of colonic flora composition are shown in Figure 4A–C. At the genus level, *Lactobacillus*, *Anaerovibrio*, *Prevotella*, *Streptococcus* and *Megasphaera* accounted for a higher proportion. In contrast, at the species level, *Lactobacillus_johnsonii*, *Streptococcus_alactolyticus*, *Megasphaera_elsdenii* and *Metagenome* accounted for a higher ratio, and adding NBAs increased the abundance of *Lactobacillus_johnsonii*. In the phylum to -genus level LEfSe analysis, ten biomarkers with significant differences were identified. *Oscillospiraceae*, Streptococcaceae, Streptococcus, Streptococcus_alactolyticus, and *UCG_002* were significantly enriched in the control group. In the 180 mg/kg NBAs group, *Lactobacillaceae*, *Lactobacillus*, *Lactobacillus_johnsonii*, *Dialister* and *Dialister_succinatiphilus* were enhanced considerably.

### 3.7. Effect of NBAs on KEGG Pathways of Colonic Flora

**Figure 5:**
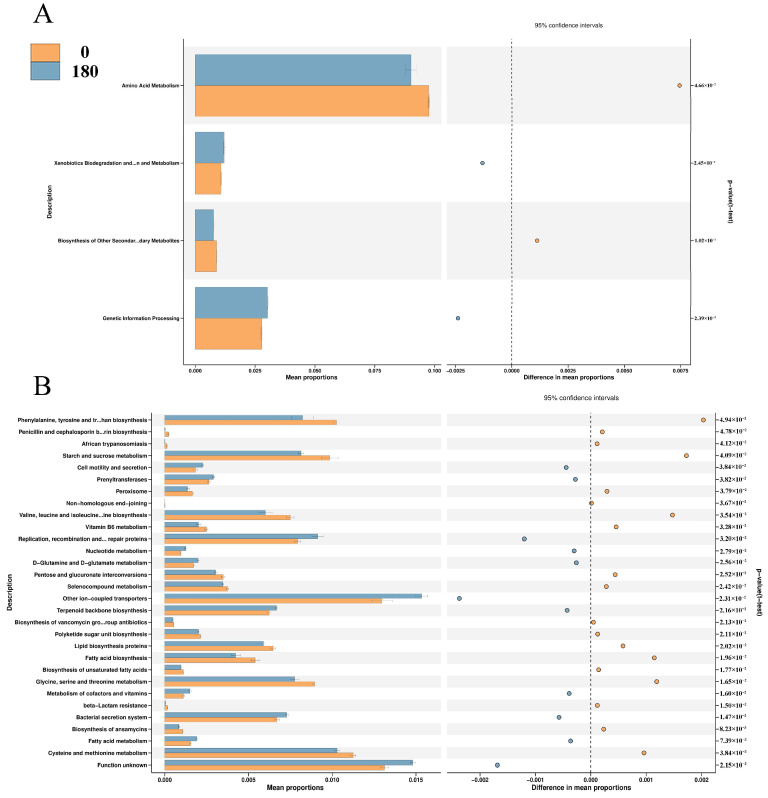
(**A**) Effects of NBAs on KEGG_level 2 pathways between groups. (**B**) Effect of NBAs on the KEGG_level 3 pathways between groups. *n* = 4.

Figure 5A,B shows the effect of NBAs on KEGG_level 2 and KEGG_level 3 pathways between groups. PICRUSt 2 can predict the metabolic capacity of colon flora, and we found that NBAs mainly affect the relative abundance of genes related to metabolic pathways such as Amino Acid Metabolism, Biosynthesis of Other Secondary Metabolites, Genetic Information Processing and Xenobiotics Biodegradation and Metabolism. Adding NBAs also significantly increased the relative abundance of genes associated with many metabolic pathways in KEGG_level 3, including D−Glutamine and D−glutamate metabolism, Fatty acid metabolism, Bacterial secretion system, Metabolism of cofactors and vitamins, Nucleotide metabolism, Prenyltransferases, Replication, recombination and repair proteins and Cell motility and secretion.

### 3.8. Effect of NBAs on SCFAs Content in Ileum

The effect of adding NBAs on colonic SCFAs content is shown in Table 4. NBAs linearly and quadratically increased the contents of propionic acid and isovaleric acid in colon contents (*p* < 0.05), and also quadratically increased the contents of acetic acid, isobutyric acid, butyric acid and valeric acid (*p* < 0.05).

### 3.9. Regression Analysis

As can be seen in Table 5, the growth performance, nutrient digestibility and blood biochemistry of weaned piglets were significantly positively correlated with the addition of NBAs. FW, ADG, ADFI and OM increased with increasing NBA supplemental level, but blood biochemical indexes such as CREA, BUN and BUN/CREA were also increased. In this study, adding 180 mg/kg of NBAs increased the content of BUN and CREA in the blood; therefore, the addition of NBAs should not exceed 180 mg/kg.

## 4. Discussion

As an intuitive indicator, growth performance reflects an animal’s growth rate. The digestibility of nutrients can reflect the absorption efficiency of nutrients in the body. In this study, the growth performance and digestibility of nutrients in piglets were closely related to NBAs, which directly reflects whether the addition of NBAs is beneficial to the growth and development of piglets. In previous studies, feeding bile acids has achieved good results, especially in poultry and pigs. Adding 80 mg/kg BAs significantly increased the digestibility of EE and GE in broilers while decreasing F/G [18]. This result was further validated in another study in which 40–80 mg/kg BAs were added [19]. In a study on pigs, feeding BAs extracted from pig bile paste increased ADG and the digestibility of EE, and GE in piglets [20]. In this study, the addition of NBAs increased the digestibility of EE and OM and increased the ADG and ADFI of piglets.

To explore whether adding NBAs damages the liver and kidneys, we examined blood biochemical markers that reflect liver and kidney health. Indicators reflecting liver bilirubin metabolism and cholestasis include TBIL, TBA, ALP and GGT. TBIL reflects bilirubin metabolism, TBA reflects hepatocyte damage, and ALP reflects cholestasis. GGT is involved in glutathione metabolism and can reflect whether the liver is diseased. BUN and CREA are important indicators often used to assess kidney function, and their elevated levels in plasma may mean that kidney function has been damaged to a certain extent. HCA and HDCA are hydrophilic BAs [21,22], while CDCA is a hydrophobic BA. Hydrophilic BAs generally have no negative effects on the body. At the same time, hydrophobic CDCA has also been shown to be beneficial to the body [23]. In this study, adding NBAs did not affect TBIL, TBA, ALP, and GGT. This further indicates that the addition of NBAs composed of HCA, HDCA and CDCA does not negatively affect the liver of piglets. However, the addition of NBAs increased the levels of BUN and CREA in plasma. The increase in BUN and CREA content indicated that NBA supplementation had a certain effect on the kidneys of piglets. In a survey of broiler chickens, it was found that adding BAs at more than ten times the standard dose still did not affect BUN and CREA [24], while in another study on piglets, adding BAs elevated BUN levels [6]. This difference in results may be due to species differences. In this study, the addition of 180 mg/kg NBAs increased the levels of BUN and CREA in the plasma of piglets, and the specific reasons for the increase still need to be further explored.

The duodenum and jejunum of piglets have essential roles in absorbing nutrients. To investigate whether the addition of NBAs promotes the development of the small intestine in weaned piglets, we examined the CD and VH in the jejunum and ileum. The villi structure in the small intestine can significantly improve the contact area with nutrients and promote their absorption. In this study, the increase in VH in the jejunum and ileum of piglets means that the contact area of the intestinal tract with nutrients increases, which is more conducive to nutrient absorption. Therefore, piglets’ growth performance and nutrient digestibility may be related to increased intestinal VH and VCR. The surface of each villus in the small intestine is a layer of columnar epithelial cells, which are characterized by rapid renewal. The rapid regeneration of small intestine epithelium mainly depends on the differentiation and migration of crypt stem cells [25]. In this study, the addition of NBAs did not significantly affect the ileum CD compared with the control group. An in vitro study found that BAs could stimulate intestinal epithelial cell proliferation [26], while regular or abnormal intestinal epithelial cell proliferation was also associated with altered microbiota [27]. This suggests that various factors may cause elevated intestinal VH and VCR.

To explore whether adding NBAs promotes the synthesis and secretion of BAs, we examined the expression of genes related to the enterohepatic circulation of BAs. The enterohepatic cycle of BAs includes BA biosynthesis, transport and metabolism. *FXR* is a crucial BA receptor that regulates various metabolic processes in the body. *FXR* in the liver can regulate the transcription of the BA synthesis gene *CYP7A1* through *SHP* [28], and *FGFR4* formed after fibroblast growth factor 19 (*FGF19*) produced by *FXR* enters the liver can also regulate the expression of *CYP7A1*. *CYP7A1* (a rate-limiting enzyme used in BA synthesis) and *CYP27A1* are both enzymes involved in BA synthesis [29]. After BA synthesis, it enters the intestine via *BSEP*, and then the apical-sodium-dependent BA transporter (*ASBT*) in the ileum actively reabsorbs BAs and enters hepatocytes via *NTCP*, a process called enterohepatic circulation [30,31]. In general, inhibition of *FXR* in the liver results in the expression of BA synthase. In this study, adding NBAs decreased FXR expression in the liver and ileum. Both HCA and HDCA in NBAs are *FXR* antagonists [32], which seems to explain the inhibition of *FXR*. *FXR* in the liver can regulate the expression of *NTCP* [33], while *NTCP* enables BAs to enter the liver from the blood. In this study, the increase in the relative mRNA abundance of *NTCP* may promote BAs entering the liver from the intestine. It has been reported that the addition of NBAs can switch the synthesis of BAs from the classical pathway to the alternative pathway and promote the expression of BA synthase in the alternative pathway [34]. The results of this study showed that the addition of NBAs increased the expression of *CYP27A1* in the alternative pathway, which mainly produced HCA in piglets, which is a kind of NBA, indicating that the addition of NBAs positively regulated the ability of piglets to produce NBAs. The above results indicate that the addition of NBAs not only increases the expression of *CYP27A1*, but also promotes the formation of NBAs and increases the proportion of NBAs in the body, thus improving body health. In addition, NBAs inhibited the expression of *FXR* in the liver, increased the expression of *NTCP*, and promoted the entry of BAs from the blood into the liver.

The gastrointestinal microbiota is the most densely populated natural ecosystem, consisting of more than 10^14^ bacterial cells [35]. Most BAs (about 95%) are absorbed at the end of the ileum and participate in enterohepatic circulation. BAs that do not enter enterohepatic circulation can selectively regulate intestinal flora after entering the large intestine by stimulating bacterial growth through BA metabolic enzymes and inhibiting the growth of BA-sensitive bacteria [21]. BAs can also be produced by microorganisms present in the distal ileum and large intestine through uncoupling, dehydrogenation and dehydroxylation reactions that produce secondary BAs [36]. Intestinal flora can also transmit signals along the gut–liver axis through other metabolites, influencing host feeding and energy metabolism [37]. 16S rDNA tests found that NBAs reduced the abundance of *Streptococcus_alactolyticus* and *Oscillospiraceae_UCG_002* in the colon, while *Lactobacillus_johnsonii* and *Dialister_succinatiphilus* were enriched significantly. Adding NBAs greatly affected the relative abundance of *Streptococcus_alactolyticus* and *Lactobacillus_johnsonii* strains, and this change also aroused our concern. In the control group, *Streptococcus_alactolyticus* was more abundant than *Lactobacillus_johnsonii*, but when NBAs were added, *Streptococcus_alactolyticus* almost disappeared from the colon, while *Lactobacillus_johnsonii* colonized in large numbers. It has been reported that *Streptococcus_alactolyticus* is a bacterial species that produces lactic acid [38], and *Lactobacillus* is also a bacterial species that produces lactic acid, which indicates that there is a specific competitive relationship between the two strains. Bile brine hydrolysase (BSH) is involved in the metabolism of BAs in the gut. At the same time, *Lactobacillus* often contain several different BSHs, enabling *Lactobacillus* to occupy the intestinal host niche and making *Lactobacillus* a rich source of BSH diversity. In addition, *Lactobacillus_johnsonii* has a high tolerance to BAs and acidic pH [39]. *Lactobacillus_johnsonii* may participate in the metabolism of BAs through BSH, and the proliferation of BAs and acidic pH in the intestine produce a large amount of lactic acid, which further inhibits the proliferation of *Streptococcus_alactolyticus*. *Lactobacillus* is a recognized probiotic that can reduce the risk of infection by inhibiting pathogenic bacteria reproduction, stimulating mucin secretion, or enhancing tight junction function [40,41]. In addition to the above two bacteria, adding NBAs also had specific effects on *Oscillospiraceae_UCG_002* and *Dialister_succinatiphilus*, but the content of these two bacteria in the intestine was relatively low. The above results show that the NBAs promoted the proliferation of *Lactobacillus_Johnsonii* after entering the intestine, causing *Lactobacillus_Johnsonii* to become the dominant bacterial species in the colon flora. *Lactobacillus_Johnsonii* and NBAs further inhibited the addition of other bacteria, thus improving the intestinal flora structure. Gut microbiota can synthesize essential amino acids, regulate fat metabolism, and activate the immune system [42,43]. According to the KEGG pathway analysis of colon flora, the colonic flora supplemented with NBAs increased the relative abundance of genes related to Xenobiotics Biodegradation and Metabolism and Genetic Information Processing metabolic pathways. This indicates that NBAs not only improve intestinal flora and promote *Lactobacillus_johnsonii* colonization, but also improve body metabolism through intestinal flora, especially by increasing the relative abundance of genes related to genetic information processing and biodegradation metabolism.

SCFAs are essential metabolites produced by intestinal microbiota, among which acetic acid, propionic acid and butyric acid account for 90% of SCFAs produced by intestinal microbiota [44]. In the detection of intestinal flora, we found that adding NBAs increased the relative abundance of *Lactobacillus* and *Dialister*, and both *Lactobacillus* and *Dialister* could increase the content of SCFAs [45,46]. *Lactobacillus* can use carbohydrates in the gut to produce lactic acid. Lactic acid has various metabolic and regulatory properties and is capable of serving as an energy source for cell renewal [47]. Other gut bacteria can also use lactic acid to convert propionic acid or butyric acid further [48], increasing the amount of SCFAs in the gut. SCFAs produced by intestinal bacteria have a variety of roles, including providing nutrients to intestinal epithelial cells, promoting intestinal development, and regulating glucose and lipid metabolism [49,50,51]. The results of this study show that NBAs first improve intestinal flora, and then intestinal flora further increases the content of SCFAs in the colon. The increase in SCFAs content can provide nutrition for the intestinal epithelium, while SCFAs and NBAs work together to promote intestinal development.

## 5. Conclusions

Adding NBAs can improve piglets’ growth performance, improve EE and OM digestibility, and promote the development of the small intestine, especially the VH of the jejunum and ileum. Adding NBAs also promotes the absorption of BAs from the gut into the liver, activating alternative pathways and improving the ability to synthesize NBAs. In addition, NBAs also improved the intestinal flora, specifically increasing the abundance of *Lactobacillus_johnsonii*, and the intestinal flora further improved the body’s metabolism, increasing the content of SCFAs in the colon. Adding NBAs can improve the growth and development of piglets after weaning and can actively improve their intestinal flora. This study also serves as a reference for the actual production of piglets fed with NBAs.

## Figures and Tables

**Table 1:** Effects of NBAs on growth performance of piglets.

Items	NBAs Addition (mg/kg)					*p*-Value		
	0	60	120	180	SEM	ANOVA	Linear	Quadratic
IW, kg	10.13	10.17	10.13	10.05	0.068	0.97	0.69	0.78
FW, kg	22.95 ^a^	24.63 ^ab^	23.98 ^a^	26.28 ^b^	0.42	0.027	<0.01	0.93
ADFI, kg	0.91 ^a^	0.93 ^ab^	0.93 ^ab^	1.07 ^b^	0.025	0.078	0.028	0.42
ADG, kg	0.45 ^a^	0.52 ^ab^	0.49 ^a^	0.58 ^b^	0.015	0.023	<0.01	0.98
F/G	2.01	1.82	1.89	1.85	0.034	0.24	0.20	0.19

Different superscript letters indicate significant differences (*p* < 0.05). IW: Initial weight, FW: Final weight, ADG: Average daily gain, ADFI: Average daily feed intake, F/G: ADFI/ADG. *n* = 6.

**Table 2:** Effect of NBAs on nutrient digestibility in piglets.

Items	NBAs Addition (mg/kg)					*p*-Value		
	0	60	120	180	SEM	ANOVA	Linear	Quadratic
GE, %	83.29	83.45	83.14	82.20	0.50	0.84	0.44	0.70
CP, %	77.82	78.76	77.06	78.29	0.57	0.78	0.96	0.90
EE, %	72.46 ^a^	73.76 ^a^	75.84 ^b^	76.14 ^b^	0.48	<0.01	<0.01	0.14
CF, %	81.21	81.54	81.20	81.86	0.32	0.88	0.58	0.86
OM, %	80.38	81.68	81.73	81.93	0.26	0.13	0.04	0.14

Different superscript letters indicate significant differences (*p* < 0.05). OM: Organic matter, CP: Crude protein, EE: Ether extract, GE: Gross energy, CF: Crude fiber. *n* = 5.

**Table 3:** Effects of NBAs on blood biochemistry of piglets.

Items	NBAs Addition (mg/kg)					*p*-Value		
	0	60	120	180	SEM	ANOVA	Linear	Quadratic
TBA (μmol/L)	11.75	23.20	11.88	21.98	2.18	0.088	0.34	0.76
TBIL (μmol/L)	0.72	1.19	0.61	0.60	0.13	0.45	0.77	0.66
ALP (IU/L)	246.18	256.28	190.33	232.20	12.18	0.24	0.34	0.44
GGT (IU/L)	32.18	34.20	34.93	36.03	1.35	0.82	0.33	0.74
BUN (mmol/L)	1.76	1.97	2.53	2.75	0.16	0.065	<0.01	0.61
CREA (μmol/L)	61.30	63.48	68.28	70.03	1.60	0.180	0.024	0.62
BUN/CREA	0.029	0.031	0.041	0.040	0.0022	0.11	0.025	0.37

TBA: Total BAs, TBIL: Total bilirubin, ALP: Alkaline phosphatase, GGT: Gamma-Glutamyl Transferase, BUN: Urea nitrogen, CREA: Creatinine, BUN/CREA: Urea nitrogen/Creatinine. *n* = 5.

**Table 4:** Effects of NBAs on SCFAs content in ileum.

Items (mmol/kg)	NBAs Addition (mg/kg)					*p*-Value		
	0	60	120	180	SEM	ANOVA	Linear	Quadratic
Acetic acid	84.42 ^a^	115.45 ^c^	105.81 ^bc^	100.97 ^b^	3.12	<0.01	0.16	<0.01
Propionic acid	56.36 ^a^	68.99 ^b^	65.61 ^b^	66.43 ^b^	1.42	<0.01	0.03	<0.01
Isobutyric acid	2.91 ^a^	4.00 ^c^	3.69 ^bc^	3.57 ^b^	0.11	<0.01	0.084	<0.01
Butyric acid	23.23 ^a^	35.55 ^c^	30.46 ^b^	28.74 ^b^	1.26	<0.01	0.33	<0.01
Isovaleric acid	4.17 ^a^	5.85 ^b^	5.83 ^b^	5.45 ^b^	0.21	<0.01	0.042	<0.01
Valeric acid	7.18 ^a^	11.89 ^c^	10.06 ^bc^	8.62 ^ab^	0.50	<0.01	0.59	<0.01

Different superscript letters indicate significant differences (*p* < 0.05). *n* = 5.

**Table 5:** NBAs regression analysis of piglets related indexes.

Items (Y)	Correlation
FW	y = 4.28 × 10^−5^x^2^ + 7.87 × 10^−3^x + 23.21
ADG	y = 1.82 × 10^−6^x^2^ + 2.46 × 10^−4^x + 0.47
ADFI	y = 8.02 × 10^−6^x^2^ −6.28 × 10^−4^x + 0.92
EE	y = −6.91 × 10^−5^x^2^ + 0.03x + 72.33
OM	y = 7.83 × 10^−3^x + 80.73
BUN	y = 1.04 × 10^−6^x^2^ + 5.7 × 10^−3^x + 1.73
CREA	y = 0.05x + 61.12
BUN/CREA	y = 7.17 × 10^−5^x + 0.03

According to the significant difference (*p* < 0.05), the regression equations of bile acids on growth performance, nutrient digestibility and blood biochemistry of weaned piglets were established.

## Data Availability

All data generated or analyzed during this study are available from the corresponding author upon reasonable request.

## Supplementary Materials

The following supporting information can be downloaded at: https://www.mdpi.com/article/10.3390/ani13213380/s1, Table S1: Basic diet ingredients and nutrient levels; Table S2: Sequences of primers used for qPCR.

## References

[1] Lewis D.S., Oren S., Wang X., Moyer M.L., Beitz D.C., Knight T.J., Mott G.E. (2000). Developmental changes in cholesterol 7alpha- and 27-hydroxylases in the piglet. J. Anim. Sci..

[2] Krogdahl A. (1985). Digestion and Absorption of Lipids in Poultry. J. Nutr..

[3] Poudel P., Samuel R., Levesque C., St-Pierre B. (2022). Investigating the effects of peptide-based, MOS and protease feed additives on the growth performance and fecal microbial composition of weaned pigs. J. Anim. Sci. Biotechnol..

[4] Zheng X., Chen T., Zhao A., Ning Z., Kuang J., Wang S., You Y., Bao Y., Ma X., Yu H. (2021). Hyocholic acid species as novel biomarkers for metabolic disorders. Nat. Commun..

[5] Yang B., Huang S., Zhao G., Ma Q. (2022). Dietary supplementation of porcine bile acids improves laying performance, serum lipid metabolism and cecal microbiota in late-phase laying hens. Anim. Nutr..

[6] Liu Y., Azad M.A.K., Zhu Q., Yu Z., Kong X. (2022). Dietary bile acid supplementation alters plasma biochemical and hormone indicators, intestinal digestive capacity, and microbiota of piglets with normal birth weight and intrauterine growth retardation. Front. Microbiol..

[7] Haeusler R.A., Pratt-Hyatt M., Welch C.L., Klaassen C.D., Accili D. (2012). Impaired Generation of 12-Hydroxylated Bile Acids Links Hepatic Insulin Signaling with Dyslipidemia. Cell Metab..

[8] Hylemon P.B., Zhou H., Pandak W.M., Ren S., Gil G., Dent P. (2009). Bile acids as regulatory molecules. J. Lipid Res..

[9] Joyce S.A., MacSharry J., Casey P.G., Kinsella M., Murphy E.F., Shanahan F., Hill C., Gahan C.G.M. (2014). Regulation of host weight gain and lipid metabolism by bacterial bile acid modification in the gut. Proc. Natl. Acad. Sci. USA.

[10] Kiriyama Y., Nochi H. (2019). The Biosynthesis, Signaling, and Neurological Functions of Bile Acids. Biomolecules.

[11] Bai G., He W., Yang Z., Fu H., Qiu S., Gao F., Shi B. (2019). Effects of different emulsifiers on growth performance, nutrient digestibility, and digestive enzyme activity in weanling pigs1. J. Anim. Sci..

[12] Cai J., Rimal B., Jiang C., Chiang J.Y., Patterson A.D. (2022). Bile acid metabolism and signaling, the microbiota, and metabolic disease. Pharmacol. Ther..

[13] Joyce S.A., Gahan C.G. (2016). Bile Acid Modifications at the Microbe-Host Interface: Potential for Nutraceutical and Pharmaceutical Interventions in Host Health. Annu. Rev. Food Sci. Technol..

[14] Qiu Y., Liu S., Hou L., Li K., Wang L., Gao K., Yang X., Jiang Z. (2021). Supplemental Choline Modulates Growth Performance and Gut Inflammation by Altering the Gut Microbiota and Lipid Metabolism in Weaned Piglets. J. Nutr..

[15] Sorrentino G., Perino A., Yildiz E., El Alam G., Bou Sleiman M., Gioiello A., Pellicciari R., Schoonjans K. (2020). Bile Acids Signal via TGR5 to Activate Intestinal Stem Cells and Epithelial Regeneration. Gastroenterology.

[16] Gao Y., Meng Q., Qin J., Zhao Q., Shi B. (2023). Resveratrol alleviates oxidative stress induced by oxidized soybean oil and improves gut function via changing gut microbiota in weaned piglets. J. Anim. Sci. Biotechnol..

[17] Bai G., Jiang X., Qin J., Zou Y., Zhang W., Teng T., Shi B., Sun H. (2022). Perinatal exposure to glyphosate-based herbicides impairs progeny health and placental angiogenesis by disturbing mitochondrial function. Environ. Int..

[18] Geng S., Zhang Y., Cao A., Liu Y., Di Y., Li J., Lou Q., Zhang L. (2022). Effects of Fat Type and Exogenous Bile Acids on Growth Performance, Nutrient Digestibility, Lipid Metabolism and Breast Muscle Fatty Acid Composition in Broiler Chickens. Animals.

[19] Lai W., Huang W., Dong B., Cao A., Zhang W., Li J., Wu H., Zhang L. (2018). Effects of dietary supplemental bile acids on performance, carcass characteristics, serum lipid metabolites and intestinal enzyme activities of broiler chickens. Poult. Sci..

[20] Cao A.Z., Lai W.Q., Zhang W.W., Dong B., Lou Q.Q., Han M.M., He D.T., Gai X.R., Sun Y.B., Zhang L.Y. (2021). Effects of porcine bile acids on growth performance, antioxidant capacity, blood metabolites and nutrient digestibility of weaned pigs. Anim. Feed Sci. Tech..

[21] Guo X., Okpara E.S., Hu W., Yan C., Wang Y., Liang Q., Chiang J.Y.L., Han S. (2022). Interactive Relationships between Intestinal Flora and Bile Acids. Int. J. Mol. Sci..

[22] Sato H., Macchiarulo A., Thomas C., Gioiello A., Une M., Hofmann A.F., Saladin R., Schoonjans K., Pellicciari R., Auwerx J. (2008). Novel Potent and Selective Bile Acid Derivatives as TGR5 Agonists: Biological Screening, Structure−Activity Relationships, and Molecular Modeling Studies. J. Med. Chem..

[23] Yoshio A., Akira A., Hiromichi B., Kouhei Y., Hisakazu D., Yasunobu K., Akihiko H., Yoshihide F. (2003). The cytotoxicity of hydrophobic bile acids is ameliorated by more hydrophilic bile acids in intestinal cell lines IEC-6 and Caco-2. Oncol. Rep..

[24] Lai W., Cao A., Li J., Zhang W., Zhang L. (2018). Effect of High Dose of Bile Acids Supplementation in Broiler Feed on Growth Performance, Clinical Blood Metabolites, and Organ Development. J. Appl. Poult. Res..

[25] Delgado M.E., Grabinger T., Brunner T. (2016). Cell death at the intestinal epithelial front line. FEBS J..

[26] Ji C., Xie X., Yin J., Qi W., Chen L., Bai Y., Wang N., Zhao D., Jiang X., Jiang H. (2017). Bile acid receptor TGR5 overexpression is associated with decreased intestinal mucosal injury and epithelial cell proliferation in obstructive jaundice. Transl. Res..

[27] Dossa A.Y., Escobar O., Golden J., Frey M.R., Ford H.R., Gayer C.P. (2016). Bile acids regulate intestinal cell proliferation by modulating EGFR and FXR signaling. Am. J. Physiol.-Gastr. L..

[28] Chiang J.Y.L. (2009). Bile acids: Regulation of synthesis. J. Lipid Res..

[29] Perino A., Demagny H., Velazquez-Villegas L., Schoonjans K. (2021). Molecular physiology of bile acid signaling in health, disease, and aging. Physiol. Rev..

[30] Ticho A.L., Malhotra P., Dudeja P.K., Gill R.K., Alrefai W.A. (2019). Intestinal Absorption of Bile Acids in Health and Disease. Compr. Physiol..

[31] Schneider K.M., Albers S., Trautwein C. (2018). Role of bile acids in the gut-liver axis. J. Hepatol..

[32] Zhou J., Li C., Liu Y., Lin S., Wang Y., Xie C., Nan F. (2022). Discovery of 9,11-Seco-Cholesterol Derivatives as Novel FXR Antagonists. ACS Omega.

[33] Ito K., Okumura A., Takeuchi J.S., Watashi K., Inoue R., Yamauchi T., Sakamoto K., Yamashita Y., Iguchi Y., Une M. (2021). Dual Agonist of Farnesoid X Receptor and Takeda G Protein-Coupled Receptor 5 Inhibits Hepatitis B Virus Infection In Vitro and In Vivo. Hepatology.

[34] Kuang J., Wang J., Li Y., Li M., Zhao M., Ge K., Zheng D., Cheung K.C.P., Liao B., Wang S. (2023). Hyodeoxycholic acid alleviates non-alcoholic fatty liver disease through modulating the gut-liver axis. Cell Metab..

[35] Ridlon J.M., Alves J.M., Hylemon P.B., Bajaj J.S. (2013). Cirrhosis, bile acids and gut microbiota: Unraveling a complex relationship. Gut Microbes.

[36] Ridlon J.M., Kang D.J., Hylemon P.B. (2006). Bile salt biotransformations by human intestinal bacteria. J. Lipid Res..

[37] Ringseis R., Gessner D.K., Eder K. (2020). The Gut-Liver Axis in the Control of Energy Metabolism and Food Intake in Animals. Annu. Rev. Anim. Biosci..

[38] Salminen S., Deighton M. (1992). Lactic acid bacteria in the gut in normal and disordered states. Dig. Dis..

[39] Davoren M.J., Liu J., Castellanos J., Rodríguez-Malavé N.I., Schiestl R.H. (2019). A novel probiotic, *Lactobacillus johnsonii 456*, resists acid and can persist in the human gut beyond the initial ingestion period. Gut Microbes.

[40] Mack D.R., Ahrne S., Hyde L., Wei S., Hollingsworth M.A. (2003). Extracellular MUC3 mucin secretion follows adherence of *Lactobacillus* strains to intestinal epithelial cells in vitro. Gut.

[41] Seth A., Yan F., Polk D.B., Rao R.K. (2008). Probiotics ameliorate the hydrogen peroxide-induced epithelial barrier disruption by a PKC- and MAP kinase-dependent mechanism. Am. J. Physiol.-Gastrointest. Liver Physiol..

[42] Hooper L. (2004). Bacterial contributions to mammalian gut development. Trends Microbiol..

[43] Hooper L.V., Gordon J.I. (2001). Commensal Host-Bacterial Relationships in the Gut. Science.

[44] XiaoFeng C., Xiangqi C., Xiaoqiang T. (2020). Short-chain fatty acid, acylation and cardiovascular diseases. Clin. Sci..

[45] He Q., Zou T., Chen J., He J., Jian L., Xie F., You J., Wang Z. (2021). Methyl-Donor Micronutrient for Gestating Sows: Effects on Gut Microbiota and Metabolome in Offspring Piglets. Front. Nutr..

[46] Cheng Y., Liu J., Ling Z. (2022). Short-chain fatty acids-producing probiotics: A novel source of psychobiotics. Crit. Rev. Food Sci. Nutr..

[47] Koh A., De Vadder F., Kovatcheva-Datchary P., Bäckhed F. (2016). From Dietary Fiber to Host Physiology: Short-Chain Fatty Acids as Key Bacterial Metabolites. Cell.

[48] Flint H.J., Scott K.P., Louis P., Duncan S.H. (2012). The role of the gut microbiota in nutrition and health. Nat. Rev. Gastroenterol. Hepatol..

[49] Zhou H., Yu B., Sun J., Liu Z., Chen H., Ge L., Chen D. (2021). Short-chain fatty acids can improve lipid and glucose metabolism independently of the pig gut microbiota. J. Anim. Sci. Biotechnol..

[50] Zhou H., Sun J., Ge L., Liu Z., Chen H., Yu B., Chen D. (2020). Exogenous infusion of short-chain fatty acids can improve intestinal functions independently of the gut microbiota. J. Anim. Sci..

[51] Parada Venegas D., De la Fuente M.K., Landskron G., González M.J., Quera R., Dijkstra G., Harmsen H.J.M., Faber K.N., Hermoso M.A. (2019). Short Chain Fatty Acids (SCFAs)-Mediated Gut Epithelial and Immune Regulation and Its Relevance for Inflammatory Bowel Diseases. Front. Immunol..

