# Supplementary material for: Non-12α-Hydroxylated Bile Acids Improve Piglet Growth Performance by Improving Intestinal Flora, Promoting Intestinal Development and Bile Acid Synthesis
Source: Animals (Basel). 2023 Oct 31;13(21):3380. doi: 10.3390/ani13213380 (PMC10650152; doi:10.3390/ani13213380)
Supplement: Supplementary file 1 [file animals-13-03380-s001.zip › Supplementation.docx]

**Table S1. Basic diet ingredients and nutrient levels**

| **Ingredients** | **Percentage(%)** |
| --- | --- |
| Corn | 57.80 |
| Soybean meal | 13.00 |
| Fish meal(60.2% CP) | 4.00 |
| Extruded soybean | 13.00 |
| Whey powder | 6.00 |
| Soybean oil | 2.00 |
| Dicalcium phosphate | 0.50 |
| Limestone | 0.90 |
| Nacl | 0.70 |
| Lysine | 0.60 |
| Methionine | 0.10 |
| Threonine | 0.20 |
| Tryptophan | 0.10 |
| Choline chloride | 0.10 |
| Premix^a^（1%） | 1.00 |
| Total | 100.00 |
| **Nutritional level^b^** |  |
| DE (Mcal/kg) | 3.47 |
| CP | 19.47 |
| Ca | 0.76 |
| Available phosphorus | 0.35 |
| Lys | 1.28 |
| Met | 0.36 |
| Thr | 0.77 |
| Trp | 0.27 |
| Na | 0.30 |

Note: ^a^ Provided the following per kilogram of diet: Fe 450mg, Cu 180mg, Mn 142mg, Zn 391 mg, Se 0.27 mg, I 0.45mg, VA ≥ 2060 IU, VD_3_ ≥ 3825 IU, VE ≥ 36 mg, VK_3_ ≥ 2.25 mg, VB_1_ ≥ 4.5 mg, VB_2_ ≥ 9 mg, VB_6_ ≥ 4.05 mg, VB_12_ ≥ 0.045 mg, Niacin ≥ 45 mg, Pantothenic acid ≥ 20.25 mg, Folic acid ≥ 1.8 mg, Biotin ≥ 0.225 mg.

^b^ Nutritional level were calculated value.

**Table S2. Sequences of primers used for qPCR**

| Gene | Primer sequence (5'to3') | Gen Bank No. |
| --- | --- | --- |
| GAPDH | F: TCGGAGTGAACGGATTTGGC  R: GAAGGGGTCATTGATGGCGA | NM_001206359.1 |
| FXR | F:AAGGACCGAGAGGCAGTAGAGAAG  R:GACCCAGGAGGCAGGCAAAATG | NM_001287412.1 |
| SHP | F:AGTGCTGCCTGGAGTCCTTATGG  R:TGGAACATCAGGGTTGAAGAGAATGG | XM_003127720.4 |
| FGFR4 | F:GCCTGCTGGGAACACTGTCAAG  R:AGCCTAATGCCTCCAATGCGATTC | XM_013987555.2 |
| KLB | F:TCAACATCTCCTCAATGGTCACACTG  R:AGGTCGGCGTAGTCTTGGAAGG | XM_003482367.4 |
| CYP27A1 | F:ACTCACTCTACGCCACCTTCCTC  R:GTATTCCAGCCATCCAGGTATCGC | NM_001243304.1 |
| CYP7A1 | F:TCCCTTGTCCTACCATAAAGTGTTGTG  R:GTCAATGCTTCTGTGCCCAAATGC | NM_001005352.3 |
| OATP | F:GCCAGCAGCACTAAGAGGATCAAG  R:TCTCTGTAAACTTTGCCTCAAGCGTAG | XM_021091164.1 |
| NTCP | F:CAACTTCACCCTCCCGCACAAC  R:ATGAGCAGCATGAACACCAGGATG | XM_001927695.5 |
| BSEP | F:GAGGTCGCCGCACAGATTACTAATG  R:GGATGGCTGTCTTGTATGGCTTCTC | XM_003133457.5 |
